# Comparative transcriptome provides insights into gene regulation network associated with the resistance to *Fusarium wilt* in grafted wax gourd *Benincasa hispida*

**DOI:** 10.3389/fpls.2023.1277500

**Published:** 2023-10-27

**Authors:** Baibi Zhu, Chunqiang Li, Min Wang, Jianjun Chen, Yanping Hu, Wenfeng Huang, Huifang Wang

**Affiliations:** ^1^ Institute of Vegetables, Hainan Academy of Agricultural Sciences, Haikou, Hainan, China; ^2^ Key Laboratory of Vegetable Biology of Hainan Province, Hainan Academy of Agricultural Sciences, Haikou, Hainan, China; ^3^ Institute of Tropical Bioscience and Biotechnology, Chinese Academy of Tropical Agricultural Sciences, Haikou, Hainan, China; ^4^ Institute of Plant Protection, Hainan Academy of Agricultural Sciences (Research Center of Quality Safety and Standards for Agro-Products, Hainan Academy of Agricultural Sciences), Haikou, Hainan, China

**Keywords:** wax gourd, graft, *Fusarium wilt*, resistance, gene regulation network

## Abstract

**Introduction:**

Wilt is a soil-borne disease caused by *Fusarium* that has become a serious threat to wax gourd production. Disease-resistant graft wax gourds are an effective treatment for *Fusarium* wilt. However, there are few reports on the defense mechanism of graft wax gourd against wilt diseases.

**Methods:**

In the present study, disease and growth indices were compared between grafted and original wax gourds after infection with *Fusarium*. High level of disease resistance was observed in the grafted wax gourd, with a lower disease index and low impacts on growth after infection. RNA-seq was performed to identify the differentially expressed genes (DEGs) between the adjacent treatment time points in the grafted and original wax gourds, respectively. Then a comparative temporal analysis was performed and a total of 1,190 genes were identified to show different gene expression patterns between the two wax gourd groups during *Fusarium* infection.

**Result and discussion:**

Here, high level of disease resistance was observed in the grafted wax gourd, with a lower disease index and low impacts on growth after infection. The DEG number was higher in grafted group than original group, and the enriched functional categories and pathways of DEGs were largely inconsistent between the two groups. These genes were enriched in multiple pathways, of which the mitogen-activated protein kinase (MAPK) signaling pathway enhanced the early defense response, and cutin, suberin, and wax biosynthesis signaling pathways enhanced surface resistance in grafted wax gourd in comparison to original group. Our study provides insights into the gene regulatory mechanisms underlying the resistance of grafted wax gourds to *Fusarium* wilt infection, which will facilitate the breeding and production of wilt-resistant rootstocks.

## 1 Introduction

Wax gourd (*Benincasa hispida*), also known as winter melon, ash gourd, white pumpkin, or white gourd, is an annual vegetable of the genus *Benincasa* Savi of the Cucurbitaceae family (Xie et al., 2019). It originated in the Indo-China region and is widely cultivated in India, Japan, China, and many other tropical areas, with increasing popularity in the Caribbean and the United States (Robinson and Decker-Walters, 1997). *De novo* assembly and annotation of the complete chloroplasts of wax gourds have revealed taxonomic discrepancies (Song et al., 2022). Wax gourd is one of the most common vegetables worldwide owing to its ease of cultivation, high yield, storage, and transportation resistance. Wild wax gourds have small fruits (<10 cm in length), whereas most wax gourd cultivars bear large fruits (up to 80 cm in length and weighing > 20 kg) (Han et al., 2013). The ripe and tender wax gourd fruits are edible and rich in carbohydrates, proteins, calcium, iron, potassium, vitamin C, many other essential nutrients, amino acids, and other nutrients (Grover et al., 2001). In addition, wax gourds contain large amounts of crude fiber, chlorophyll, polyphenols, flavonoids, and many other natural antioxidant compounds that have certain pharmacological health effects and can be used to treat various diseases (Gu et al., 2013). Genes related to the flavor and nutrition of wax gourds have been identified using transcriptome analysis (Xue et al., 2022). A new chromosome-level genome assembly and annotation of *B. hispida* has been reported (Luo et al., 2023).

In recent years, as the planting area of wax gourds has expanded, the replanting index has also increased. Wax gourd cultivars tend to be monocultured, and the occurrence of diseases has become increasingly prominent. Notably, *Fusarium* wilt has become a major constraint in the wax gourd industry (Yuan et al., 2003). Wax gourd *Fusarium* wilt is a soil-borne fungal disease caused by *Fusarium oxysporum* Schl. F. sp. *benincasae* occurs throughout the reproductive period of wax gourds. Once successfully colonized in soil, *Fusarium* wilt is almost impossible to eliminate (Dita et al., 2018). Consequently, old vegetable fields with many years of continuous cropping are more likely to experience critical periods, such as flowering, fruit set, and fruit expansion. The incidence of wilt is 60% or higher during critical periods such as flowering, fruiting, expansion, and even extinction (Kang et al., 2012). This has a significant effect on the yield and quality of wax gourds. The higher the temperature, the more serious the disease is, and the disease occurrence peaks at a temperature of 28–32 °C (Liu et al., 2002). Humidity is another important factor that influences the development of *Fusarium* wilt. Rain followed by Sunny days during summer promotes rapid development of the disease. Waterlogged plots, acidic soils, multiple years of continuous cropping, and increased underground pests promote disease development. Yield reduction restricts the healthy development of wax gourds (Asha et al., 2011).

Traditional methods of disease control mainly include crop rotation, chemical agents, and the selection of disease-resistant varieties; however, these methods are difficult to implement because of the constraints of land resources and supporting facilities, environmental protection, and lack of disease-resistant resources. Grafting cultivation technology can effectively improve the resistance of crops to *Fusarium* wilt by using rootstocks with high resistance or immunity (Colla et al., 2010). Graft cultivation is widely used for wax gourds, watermelons, and cucumbers (Usanmaz and Kazım, 2018; Kombo and Sari, 2019). With the development and popularity of grafting technologies, research on grafting has become necessary to solve practical problems. Researchers are currently focusing on rootstock screening, grafting methods, rootstock grafting after melon growth and development, and physiological and biochemical metabolism (Nordey et al., 2020). Elucidating the mechanism of grafting requires the integrated application of multidisciplinary techniques, such as cell biology, plant physiology and pathology, and molecular biology. However, the immune response mechanism of grafted wax gourds against *Fusarium* wilt infestation has not yet been fully elucidated (Raddatz-Mota et al., 2020). Systematic analysis of resistance genes can help reveal the metabolic pathways, signaling, and molecular regulatory networks of grafted wax gourds. By obtaining the differential genes, key disease resistance genes could be identified, which can provide the genetic resources for grafted wax gourd genetic engineering breeding against *Fusarium* wilt resistance. In this study, we used artificial inoculation of *Fusarium* wilt-resistant rootstocks to infest different resistant grafted seedlings of wax gourd on the basis of screening wilt-resistant rootstocks. Transcriptome analysis was used to explore the molecular mechanisms of wax gourd grafting to improve resistance to *Fusarium* wilt disease. This will provide important theoretical basis for solving the problem of succession barriers in wax gourd production and also provide important practical guidance for the selection and breeding of *Fusarium* wilt-resistant rootstocks for wax gourd grafting production.

## 2 Materials and methods

### 2.1 Plant material and sampling

The experimental materials used in this work were high sensitivity wax gourd self-rooted seedlings “Haiyou 8,” and a pumpkin rootstock “Haizhan 1,” provided by the Hainan Academy of Agricultural Sciences. Before the grafting process, rootstock seedlings developed the first true leave and wax gourd seedlings had two cotyledons emerged. The topplug technique was used for grafting (Zhou et al., 2010). Pericarps of self-rooted and grafted wax gourds at commodity maturity were extracted for subsequent experiments.

### 2.2 The treatment of *Fusarium wilt*

The *Fusarium wilt* strain used in this study was *Fusarium oxysporum* Schl*. F.* sp. *benincasae*, a specialized wax gourd wilt disease pathogen was provided by the Institute of Vegetable Research, Guangxi Academy of Agricultural Sciences. The pathogen inoculation and *Fusarium wilt* treatment were performed as described by Xie et al. (2011). Briefly, the strains were well maintained and transferred to potato dextrose agar medium for 7 days before inoculation. Agar disks cut from 7-day-old cultures were filtered through two layers of sterile gauze to remove any mycelial fragments and then diluted to a concentration of 1 × 10^6^ cfu/mL with sterile distilled water. When the third true leaf had emerged, the seedlings were inoculated with 10 mL of endoconidial suspension (with fungus) through slightly injured root dipping for 15 min, and then set in the nutrient cup, and kept for an additional 48 h.

### 2.3 Measurement of the resistance to *Fusarium wilt*

The growth indices of the original wax gourds (T) and grafted wax gourds (H) were measured before infection (TK and HK, respectively) and at 2 days (T2 and H2, respectively), 8 days (T3 and H3, respectively), and 12 days (T4 and H4, respectively) after infection. Further, length and width of the third true leaf, length of the plant from the base of the stem to the growing point (plant height) and main stem, and the plant stem base diameter (stem thickness) were measured. Each treatment of wax gourd was inoculated in 45 plants. The number of diseased plants at each time point was counted to calculate the disease index according to the disease level criteria (**Table S1**). Disease index = (∑disease level* number of plants at this disease level/highest disease level* total number of surveyed plants) *100. The experiment was repeated thrice. Leaves from six seedlings were harvested as one sample at each time point and three biological replicates were used for RNA-seq. The leaves were rinsed with water, immediately blotted with filter paper to remove residual water from the surface, and immediately snap-frozen in liquid nitrogen with three biological replicates.

### 2.4 RNA extraction and RNA-seq analysis

For RNA isolation and library preparation, RNA was prepared using a Qiagen RNeasy Kit (Qiagen, Valencia, CA, USA) according to the manufacturer’s instructions. Three replicates were collected from each group. Concentration and quality were determined using a Qubit2.0 fluorometer and agarose gel electrophoresis, respectively. Eukaryotic mRNA was enriched with a polyA tail using magnetic beads containing oligo (dT), and the mRNA was interrupted with buffer. Using the fragmented mRNA as a template and random oligonucleotides as primers, the first strand of cDNA was synthesized using the M-MuLV reverse transcriptase system. Subsequently, the RNA strand was degraded using RNase H and the second strand of cDNA was synthesized using dNTPs as the raw material in the DNA polymerase I system. Purified double-stranded cDNA was subjected to end repair, A-tailed addition, and sequencing. Approximately 200 bp of cDNA was screened using AMPure XP beads and amplified using PCR. The PCR products were purified using AMPure XP beads to obtain the library. cDNA libraries were sequenced using the Illumina NovaSeq 6000 platform.

Raw data were preprocessed using fastp (v0.19.3) with parameters “–n_base_limit 10 –qualified_quality_phred 20,” and clean reads were then aligned to the wax gourd reference genome (NCBI accession No. GCF_009727055.1) with HISAT2 (v2.1.0). The transcript of *Benincasa hispida* was reconstructed using StringTie (v 2.0). Gene expression levels were quantified using RSEM (v. 1.2.30). DESeq2 (v1.22.1) was used to perform differential gene expression analysis between groups. Genes with |log2foldchang| ≥ 1 and FDR < 0.05 were identified as significantly differentially expressed genes (DEG). Gene Ontology (GO) and Kyoto Encyclopedia of Genes and Genomes (KEGG) enrichment analyses were performed for DEG using ClusterProfiler (v3.10.1).

### 2.5 Temporal analysis

Temporal analysis was performed to determine the trends in gene expression patterns in multiple samples at a series of time points using a cluster approach. The DEGs were clustered using Short Time-series Expression Miner software (STEM) (Ernst and Bar-Joseph, 2006) with a maximum unit change in model profiles between time points of 1, a maximum output profile number of 20, and a minimum ratio for fold changes of DEGs of no less than 2. Clustered profiles (*P* < 0.05) were subjected to functional annotation analysis using a hypothesis test. The KEGG pathways with Qvalue ≤ 0.05 was considered as significantly enriched KEGG pathways for these significantly clustered profiles.

### 2.6 Weighted correlation network analysis

Co-expression network analysis was performed in R (v 4.0.0) using the weighted gene co-expression network analysis (WGCNA) package (v1.47) (Langfelder and Horvath, 2008). Genes with reads per kilobase per million > 0.3 were used for the WGCNA. Modules were identified using the automatic network construction function blockwiseModules with default parameters, except that the power was 20, minModuleSize was 50, and MergeCutHeight was 0.7. A module-trait relationship was also analyzed using the module eigengene, storage (1–4 for four consecutive stages), and tissue type (0 for peel and 1 for pulp).

GO and KEGG pathway enrichment analyses were performed for the genes in each module as described above. In addition, network connections among the most connected genes (topological overlap above the threshold of 0.15) for the modules were visualized using Cytoscape (v 3.7.1).

### 2.7 qRT-PCR analysis

Ten core genes in the regulatory network were selected for qRT-PCR analysis to validate their expressions. First-strand cDNA was synthesized using the PrimeScript First Strand cDNA Synthesis Kit (Takara, Japan). Primer sequences were designed using National Center for Biotechnology Information primer-BLAST (**Table S2**). qRT-PCR was performed by using PerfectStart^®^ Green qPCR SuperMix (TansGen, Beijing, China). qPCR reactions were performed using 1 μL of 1:10 diluted cDNA and qPCR mix in a final volume of 10 μL. Thermal cycling conditions were 5 min at 95°C followed by 40 cycles of 15 s at 95°C and 60 s at 60°C, and finally 5 s per step from 65°C to 95°C at a dissociation curve analysis. All reactions were performed in triplicate. The relative expression levels of the target genes were calculated as fold changes by normalization to the 18S rRNA gene. The obtained results were analyzed using 2^−ΔCT^ method.

### 2.8 Statistical analysis

Differences among growth indices were calculated using *t*-test. The Pearson correlation coefficients between the gene expression values of the transcriptome were calculated using R v3.6.3. Statistical significance was set at *P* < 0.05.

## 3 Results

### 3.1 Physiological measurement of wax gourd

**Figure 1:**
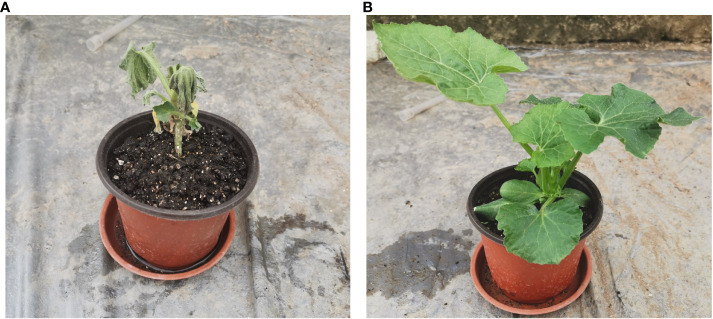
The phenotypes of original **(A)** and grafted **(B)** wax gourd after 12 days of infection.

**Figure 2:**
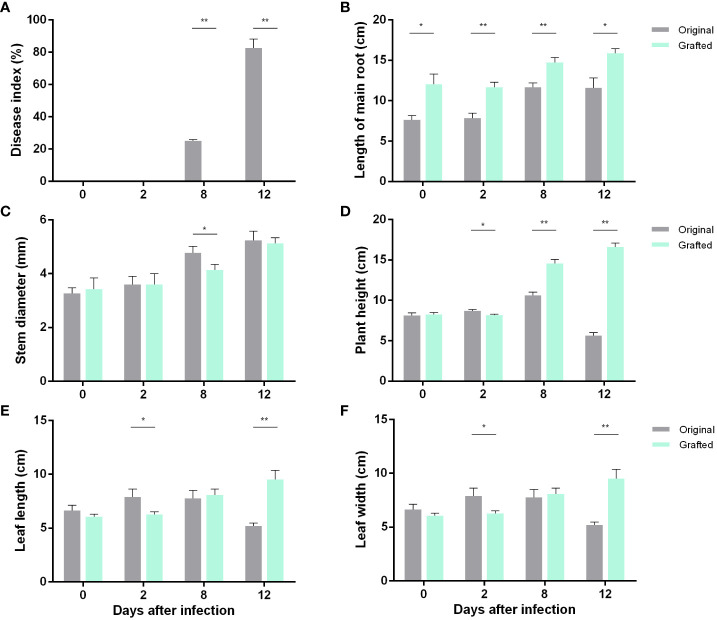
Effects of Fusarium wilt infection on the growth of wax gourd. Disease index **(A)**, length of main root **(B)**, stem diameter **(C)**, plant height **(D)**, leaf length **(E)**, leaf width **(F)**. Data are means ± SE of three replicates. Significant differences between two treatments were marked by stars (*t*-test). *, at 0.05 level; **, at 0.01 level.

The phenotypes of the original (**Figure 1A**; **Figure S1B**) and the grafted (**Figure 1B**; **Figure S1A**) wax gourds infected with *Fusarium* wilt were observed, and significant differences in the phenotypes between the two groups after 12 d of infection were seen. We measured different indicators in the two wax gourd seedlings during the infection process, as shown in **Figure 2**. In the untreated wax gourds, the disease index increased with the duration of infection; however, it had no effect on the grafted wax gourds (**Figure 2A**). Although *Fusarium* wilt affected main root growth, it was consistently lower in the original wax gourds than in the grafted wax gourds throughout the detection period (**Figure 2B**). The stem diameter of the grafted wax gourds was lower than that of the original wax gourds only 8 d after infection (**Figure 2C**). Interestingly, compared to the original wax gourd, plant height, leaf length, and leaf width in the grafted wax gourd decreased after 2 d following infection and then significantly increased after 12 d of infection (**Figures 2D–F**).

### 3.2 L transcriptome analysis of wax gourds with different resistance to *Fusarium wilt* disease

**Figure 3:**
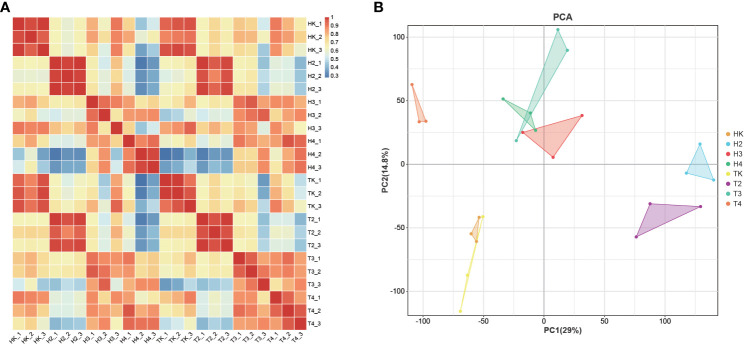
Correlation analysis between different samples in RNAseq analysis **(A)** and principal component analysis of samples **(B)**. TK: the original wax gourds before infection; HK: the grafted wax gourds before infection; T2: the original wax gourds 2 days after infection; H2: the grafted wax gourds 2 days after infection; T3: the original wax gourds 8 days after infection; H3: the grafted wax gourds 8 days after infection; T4: the original wax gourds 12 days after infection; H4: the grafted wax gourds 12 days after infection.

**Figure 4:**
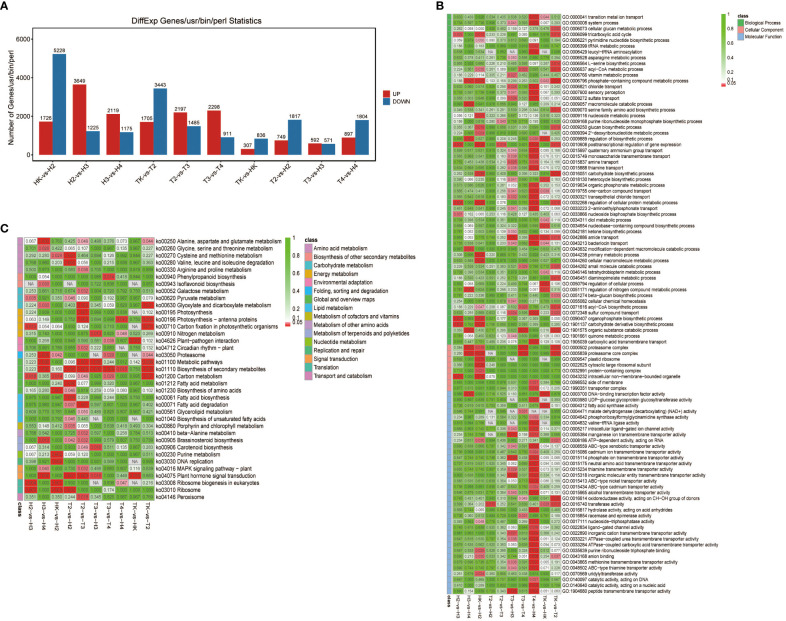
The number of DEGs **(A)** and GO **(B)**, KEGG pathway enrichment analyses **(C)** at different time points in the grafted (H) and original (T) wax gourd. TK: the original wax gourds before infection; HK: the grafted wax gourds before infection; T2: the original wax gourds 2 days after infection; H2: the grafted wax gourds 2 days after infection; T3: the original wax gourds 8 days after infection; H3: the NA, not available. grafted wax gourds 8 days after infection; T4: the original wax gourds 12 days after infection; H4: the grafted wax gourds 12 days after infection.

To obtain the gene expression profile during *Fusarium* wilt infection, we performed RNA-seq analysis of three biological replicate leaf samples from the two types of wax gourds before infection and at four time points after infection. The total raw and clean reads in each sample ranged from 4,694,039,896 to 5,949,050,525, and from 4,682,502,603 to 5,930,508,625, respectively (**Table S3**). More than 91.5% of the sequenced bases had a quality score of Q30 or higher. The total mapping rate of all samples mapped to the reference genome was between 94.51% and 97.45% (**Table S4**), indicating that the data were fully usable for subsequent analysis. The Pearson’s correlation between replicates ranged from 0.999 to 1, suggesting that the transcriptome results were reliable and stable (**Figure 3A**), as confirmed by the principal component analysis (PCA). Samples in the same groups clustered together, indicating consistency between the samples. Samples collected from different varieties were far apart, suggesting variation between the two varieties (**Figure 3B**). The DEG between the comparison groups was analyzed with |log2FC|≥1 and FDR< 0.05. The results showed that the number of DEGs at adjacent time points in the grafted wax gourds (H, resistant) was generally higher than that in the original wax gourds (T, susceptible) (**Figure 4A**; **Table S5**). The total number of DEGs in HK vs. H2 was 6,954 (1,726 upregulated and 5,228 downregulated); that in H2 vs. H3 was 4,874 (3,649 upregulated and 1,225 downregulated); and that in H3 vs. H4 was 3,294 (2,119 upregulated and 1,175 downregulated). The total number of DEGs in TK vs. T2 was 5,148 (1,705 upregulated and 3,443 downregulated), that in T2 vs. T3 was 3,682 (2,197 upregulated and 1485 downregulated), and T3 vs. T4 were 3,209 (2,298 upregulated and 911 downregulated) (**Figure 4A**; **Table S5**). Furthermore, prolonged infection with *Fusarium* wilt led to increased gene upregulation in the different wax gourd groups. These results indicate that infection with *Fusarium* wilt in rootstocks can mobilize more genes that participate in disease resistance.

We performed a functional annotation analysis of the DEGs based on the GO and KEGG databases. The GO terms were grouped into three categories: biological processes, cellular components, and molecular functions. Most DEGs produced by *Fusarium* wilt disease at T3-vs-H3 and T4-vs-H4 were grouped into molecular functions and biological processes, and HK-vs-H2 were grouped into cellular components (**Figure 4B**). Although the number of DEGs differed between the two types of wax gourds before infection and at the four time points after infection, most of these genes participated in biological processes, especially metabolic processes, according to the GO term enrichment analysis (**Figure 4B**).

KEGG pathway enrichment analyses revealed that the biosynthesis of secondary metabolites (ko01110) and metabolic pathways (ko01100) were active in most comparison groups. When comparing different infection time points in the grafted wax gourd (H, resistant) group, the DEGs were largely enriched in alanine, aspartate, and glutamate metabolism (ko00250) and glyoxylate and dicarboxylate metabolism (ko00630) pathways (**Figure 4C**). At the same time point, the DEGs between the grafted and original wax gourds were generally enriched in the plant hormone signal transduction pathway (ko04075) (**Figure 4C**). Notably, the peroxisome pathway (ko04146) related to antioxidation was only enriched by the DEGs of grafted wax gourd (T2 -vs-T3).

### 3.3 Comparison of trends in wax gourd across different stages

**Figure 5:**
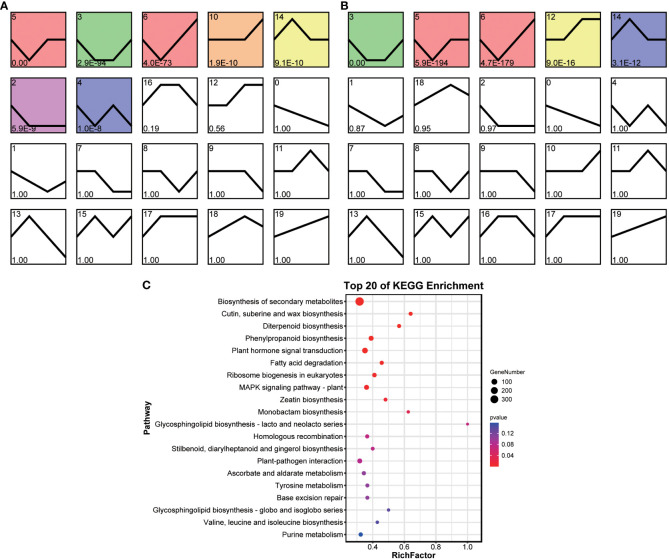
Temporal expression trends of grafted **(A)** and original **(B)** wax gourd responded to Fusarium wilt. KEGG pathway enrichment analysis for genes in trend profiles that of significant differences **(C)**. TK: the original wax gourds before infection; HK: the grafted wax gourds before infection; T2: the original wax gourds 2 days after infection; H2: the grafted wax gourds 2 days after infection; T3: the original wax gourds 8 days after infection; H3: the grafted wax gourds 8 days after infection; T4: the original wax gourds 12 days after infection; H4: the grafted wax gourds 12 days after infection.

Temporal analysis was used to estimate the gene expression patterns of the grafted and original wax gourds in response to *Fusarium* wilt. DEGs were clustered into 20 background-clustered profiles for each wax gourd, and significant profiles were selected based on p < 0.05. Of the 20 clustered profiles, seven were separately identified as significantly clustered in the grafted wax gourd, including profiles 2, 3, 4, 5, 6, 10 and 14 (**Figure 5A**), whereas the significantly clustered profiles in the original wax gourd were profiles 3, 5, 6, 12, and 14 (**Figure 5B**). There was a slight difference in the DEGs trends between the two groups of wax-gourd samples.

We further narrowed the trend changes into six modes: overall up, overall down, up to back, down to back, up to down, and down to up. We then selected genes that conformed to the six modes, but the modes were inconsistent between the two wax gourd samples, and 1,190 genes were selected (**Table S6**). These genes were significantly enriched in the biosynthesis of secondary metabolites (ko01110); cutin, suberin, and wax biosynthesis (ko00073); diterpenoid biosynthesis (ko00904); phenylpropanoid biosynthesis (ko00940); plant hormone signal transduction (ko04075); ribosome biogenesis in eukaryotes (ko03008) and the MAPK signaling pathway (ko04016) (**Figure 5C**). Further, 25 methyltransferases were found in the differential trend genes in various pathways (**Table S6**), suggesting a potential differences in methylation levels in different pathways between two wax gourds.

### 3.4 Differential regulation pathways between the original and grafted wax gourds

**Figure 6:**
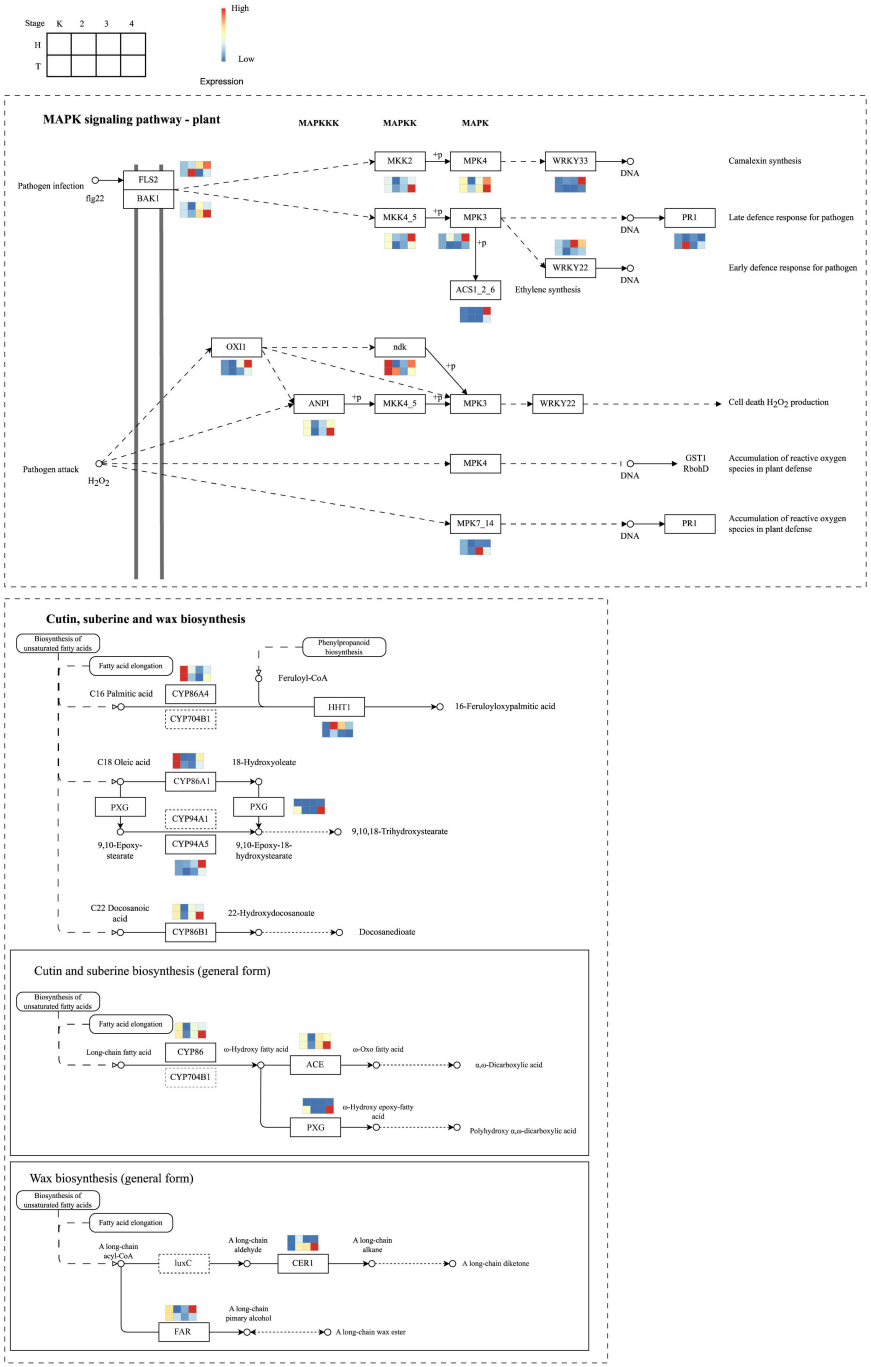
Differential regulation of MAPK signaling pathway between the original and grafted wax gourds.

According to the KEGG pathway enrichment analysis of different trend genes, the MAPK signaling pathway was differentially regulated between the original and grafted wax gourds. Within this pathway, genes, including LRR receptor-like serine/threonine-protein kinase (FLS2), mitogen-activated protein kinase kinase 4 (MKK4), mitogen-activated protein kinase 3 (MPK3), and WRKY transcription factor 22 (WRKY22), were significantly increased in grafted wax gourds (H, resistant) after the lapse of infection, whereas there were no obvious changes in the original wax gourds (T, susceptible) (**Figure 6**). This pathway corresponds to an early defense response pathway targeting pathogenic microorganisms, indicating that grafted wax gourds have a more active early defense response than the original wax gourds.

Other differentially regulated pathways are the cutin, suberin, and wax biosynthesis pathways. Key genes including CYP86A4, CYP86A1, peroxygenase (PXG), CYP94A5, CYP86B1, CYP86, fatty acid omega-hydroxy dehydrogenase (ACE), and alcohol-forming fatty acyl-CoA reductase (FAR) were downregulated in the two types of wax gourds (**Figure 6**). However, some genes in the cutin and suberin biosynthesis (general form) pathways were significantly increased after infection for 12 d in the original wax gourd (T, susceptible), including CYP86, ACE, and PXG, as well as aldehyde decarbonylase (CER1), which belongs to the wax biosynthesis (general form) pathway. Furthermore, quantitative real-time PCR (qRT-PCR) analysis of genes with high centrality scores in the networks validated the reliability of their expression patterns (**Figure S2**).

## 4 Discussion

To date, the genes responsible for the resistance of wax gourd against *Fusarium* wilt have not been identified. The molecular mechanisms underlying the interactions between wax gourds and *Fusarium* spp. is yet to be elucidated. In this study, we compared the DEGs between grafted and wilt-susceptible species. Our study provides insights into the mechanism of resistance of grafted species to *Fusarium*. In this study, we provide theoretical support for graft cultivation, which could solve the continuous crop barrier for wax gourds.

Because there is little research on wax gourd *Fusarium* wilt, especially on the grading of disease and resistance to wilt, there is no unified standard. The evaluation criteria for different melon crops differ, and there are five methods to identify resistance to melon wilt seedling inoculation: mycorrhizal soil method, embryonic root inoculation method, root irrigation method, root dipping method, and pathogenic bacterium toxin filtrate dipping method (Ren et al., 2017). Among all inoculation methods, the wounded root irrigation inoculation method has been reported the simplest and most common (Wu, 2008). When the test material is inoculated using different inoculation systems, the final plants exhibit different levels of resistance. Therefore, it is particularly important to select different concentrations and dosages for inoculation with *Fusarium* wilt bacteria. Previous studies have shown that the susceptibility index correlates well with the resistance of a material. The susceptibility index is the main indicator of plant resistance (Chen et al., 2014); therefore, the disease resistance index was used as the main measure in this experiment. In this study, the wounded root irrigation method was used to investigate wax gourd *Fusarium* wilt resistance in order to identify the best inoculation system (**Figure 1**). The results showed that the original wax gourd disease index was higher than that of the grafted species during infection with *Fusarium* wilt. During infection, the main root length trend was consistent with that of the disease index. These results indicated that the grafted wax gourds were less susceptible to *Fusarium* wilt. This result was consistent with that of a previous study (Ye et al., 2019).

To date, transcriptomic techniques have been used to study the mechanisms of intercropping *Fusarium* wilt and wax gourds; however, genes related to wilt resistance have not been reported. Further, the molecular mechanisms underlying the interactions between wax gourds and *Fusarium* wilt have not been studied extensively. In this study, we used RNA-seq to analyze gene expression profiles before and after inoculation with *Fusarium* bacteria. DEGs at adjacent time points in the grafted wax gourds were generally higher than those in the original wax gourds (**Figure 4**). Longer infection with *Fusarium* wilt led to the upregulation of more genes in different wax gourd groups. This suggests that the grafted wax gourds transferred more genes involved in disease resistance than the original wax gourds. This is because plant cells specifically recognize pathogen effectors using their own disease-resistant proteins when a pathogen invades a plant. As a part of the cell’s inherent disease resistance response, the expression of a large number of genes involved in disease resistance-related pathways is activated, leading to disease resistance and thus to biological defense against pathogenic bacteria after stress. Our results are consistent with previously reported disease resistance mechanisms in melons (Li et al., 2016; Ding et al., 2020).

In this study, the KEGG pathway enrichment analysis revealed that the metabolic pathway was active in most of the comparison groups (**Figure 4C**). These results indicate that this metabolic pathway participates in resistance to *Fusarium* wilt. This is consistent with previous findings that metabolic pathways such as phenylalanine synthesis and metabolism may be jointly involved in plant disease resistance responses (Zhang et al., 2017; Zhao, 2018). The number of DEGs associated with phytohormone signaling was reported to be higher after inoculation of susceptible varieties in comparison to the non-infected sample (Berens et al., 2017). The main phytohormone signaling pathway related to plant resistance is the jasmonic acid (JA) pathway. It has been reported that the development of resistance of Gansu peaches may be closely related to the phenolpropane metabolic pathway (Wei et al., 2011). By studying companion wheat, scientists found that disease resistance is achieved by regulating the expression of genes involved in the phenylpropanoid and lignin synthesis pathways (Xu, 2014). Previous studies have suggested that lignin protects plants by maintaining the strength and integrity of plant cell walls, which plays a major role in protection of plants from pathogenic bacteria (Lin and He, 2020).

In this study, the differential genes were mostly enriched in the MAPK signaling pathway (**Figures 5**, **6**). The MAPK cascade is an important signal transduction pathway that contains three kinases: mitogen-activated protein kinase (MAP3K), mitogen-activated protein kinase kinase (MAPKK), and mitogen-activated protein kinase MAPK (Cakir and Kilickaya, 2015). Studies have shown that MAPK plays a role in the abiotic stress response in plants. Black-seeded pumpkins recognize pathogen effectors and specific pathogen receptors via NBS-like disease resistance proteins. The MPAK signaling pathway is activated triggering the activation of NADPH oxidase, which protects against pathogens by mediating stomatal closure, increasing pyruvate levels, and reducing transpiration and programmed cell death (Ding et al., 2020). A previous study showed that *GbMPK3* in transgenic tobacco (*Nicotiana tabacum*) enhanced drought tolerance and reduced water damage (Long et al., 2014). Researchers have found that *OsMPK5* in rice (*Oryza sativa*) exhibits salt stress tolerance (Xiong and Yang, 2003). In addition, MPAK plays a role in phytohormone signaling (Shi et al., 2010; Beck et al., 2011; Danquah et al., 2013; Yu et al., 2016), plant growth and development (Kosetsu et al., 2010; Meng and Zhang, 2013), and plant disease resistance (Mao et al., 2011). This is consistent with the results of the present study.

In addition, temporal analysis showed that genes with different expression trends between the graft and original wax gourds were significantly enriched in cutin, suberin, and wax biosynthesis (**Figures 5**, **6**). Cutin is a major component of cuticle, which could protect the plant from biotic and abiotic stresses (Xin et al., 2021). Cutin and suberin are hydrophobic lipid biopolyester components of the cell walls of specialized plant tissue and cell-types, where they facilitate adaptation to terrestrial habitats (Philippe et al., 2020). In pumpkin tissues, cadmium influenced cutin, suberin and wax biosynthesis in the leaf (Han et al., 2021). Suberin and wax biosynthesis participated in sprouting in potato (Li et al., 2022). In kiwifruit and arabidopsis, CYP86 family could promote the accumulation of ω-hydroxyacids, α, ω-diacids, fatty acids and primary alcohols (Wei et al., 2020). Ω-hydroxyacids and other fatty acids participated in constructing plant cell walls, which was related with disease resistance (Graça, 2015). Fatty acid omega-hydroxy dehydrogenase (ACE), peroxygenase (PXG) as well as aldehyde decarbonylase (CER1), which belongs to the wax biosynthesis pathway. It has been reported that cutin, suberin and wax biosynthesis signaling pathway activates the defense responses in Arabidopsis (Zhang et al., 2020). This is consistent with our findings. ACE, PXG and CER1 engaged in the resistance to *Fusarium wilt* in grafted wax gourd *Benincasa hispida.* As a mixture of very-long-chain (>C20) fatty acids and their derivatives, cuticular waxes play multiple roles, from acting as a plant physical barrier to limit the entry of pathogens to functioning as cues exploited by pathogens to initiate their pre-penetration and infection processes in regulating the plant-pathogen interactions (Wang et al., 2020). It has been reported that the cuticle acts as a barrier to blight in barley spikelets (Kumar et al., 2016). In rice, wax synthesis regulator genes are significantly upregulated in blight-resistant lines (Xiao et al., 2022). Latest research showed that genetic variation in ZmWAX2 confers resistance to *Fusarium* verticillioides in maize (Ma et al., 2023).

Besides the above two pathways, we found that the peroxisome pathway (ko04146) related to antioxidation was only enriched by the DEGs of grafted wax gourd (T2 -vs-T3). This result suggests that antioxidative pathway might altered in grafted wax gourd during infection. In fact, antioxidation process was involved in resistant to *Fusarium wilt* in pine (Zhang et al., 2022), pepper (Abdelaziz et al., 2022), tomato (Narasimhamurthy et al., 2022) and chickpea (Ankati et al., 2021). Furthermore, peroxisome pathway participated in *Fusarium* resistance in banana (Cheng et al., 2019), soybean (Naeem et al., 2021) and tobacco (Wang et al., 2019). Moreover, DNA methylation might also play a role in the *Fusarium* wilt resistance in grafted wax gourd, since 25 methyltransferases were found in the genes with different expression trends between groups (**Table S6**). It has been reported that methylation related with improving stress tolerance in cucumber/pumpkin (Li et al., 2023). The methylation levels were reported to relate with wax biosynthesis in pericarp of grafting cucumber (Zhang et al., 2019). Wax biosynthesis could be resistant to *Fusarium wilt* on physical level. Therefore, the roles of epigenetic regulation in grafted wax gourd during *Fusarium* infection deserve further investigation in the future.

## 5 Conclusion

**Figure 7:**
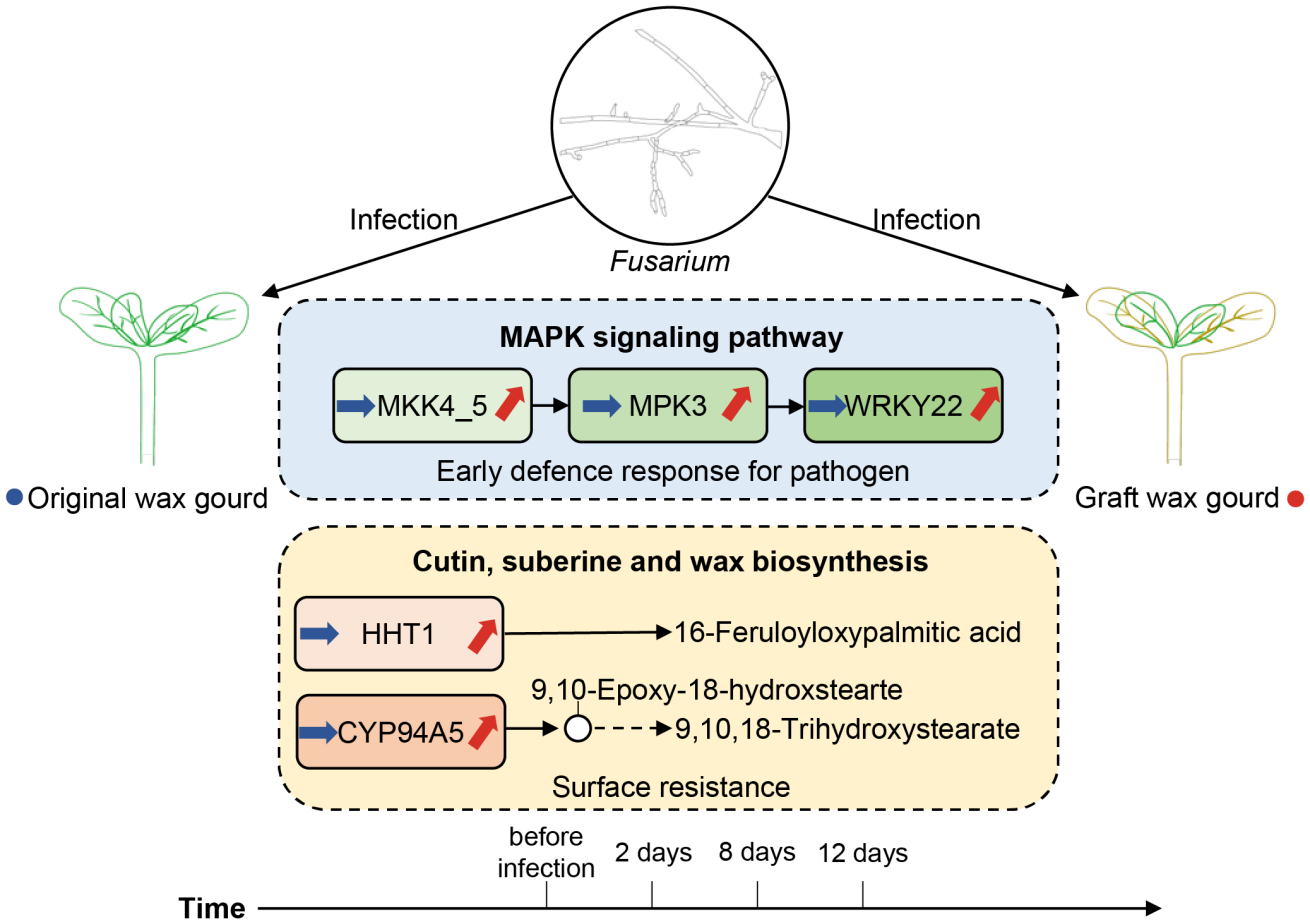
Schematic overview of the differential gene regulation mechanisms between grafted and original wax gourds in response to Fusarium wilt infection.

Currently, the demand for new varieties of high-quality wax gourds is increasing. It is necessary to use grafting methods to screen for new disease-resistant varieties of winter melons. Here, we validated high-level *Fusarium* wilt resistance in grafted wax gourds. More importantly, we used comparative temporal analysis to unveil the differences in gene expression trends between grafted and original wax gourds during the infection. In response to *Fusarium* wilt infection, the grafted wax gourd can use MAPK signaling pathway to enhance blight resistance internally, and regulate the cutin, suberin, and wax biosynthesis to enhance surface resistance simultaneously (**Figure 7**). This study investigated the molecular mechanism of wilt resistance in grafted wax gourds, which is an important guideline for the selection of wilt-resistant rootstocks and future graft production of wax gourds. Considering samples from saplings stage and investigation of mechanism of wilt resistance throughout the developmental process will strengthen present findings and is a direction for our future work.

## Data availability statement

The datasets presented in this study can be found in online repositories. The names of the repository/repositories and accession number(s) can be found below: BioProject, PRJNA962872.

## Author contributions

BZ: Conceptualization, Data curation, Methodology, Writing – original draft. CL: Software, Data curation, Writing – original draft. MW: Methodology, Writing – original draft. JC: Project administration, Supervision, Writing – original draft. YH: Investigation, Resources, Writing – original draft. WH: Validation, Visualization, Writing – original draft. HW: Funding acquisition, Writing – review & editing.

## Supplementary material

Supplementary Figure 1 The phenotypes of grafted **(A)** and original **(B)** wax gourd after 12 days of infection.
