# Supplementary figures and images for: Comparative transcriptome provides insights into gene regulation network associated with the resistance to Fusarium wilt in grafted wax gourd Benincasa hispida
Source: Front Plant Sci. 2023 Oct 27;14:1277500. doi: 10.3389/fpls.2023.1277500 (PMC10641703; doi:10.3389/fpls.2023.1277500)

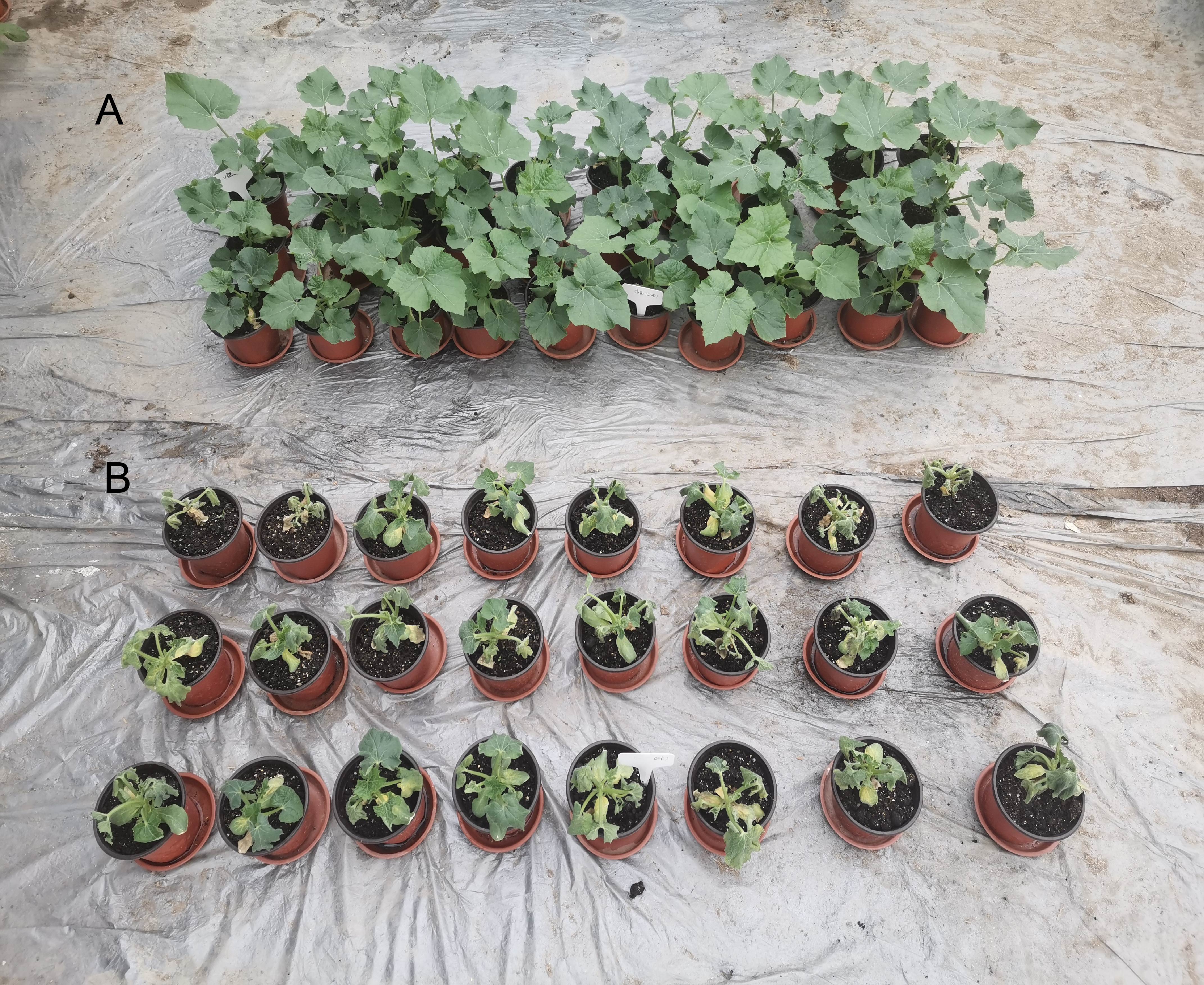

Supplement: Supplementary Figure 1 — The phenotypes of grafted (A) and original (B) wax gourd after 12 days of infection. [file Image_1.jpeg]

RNA-seq

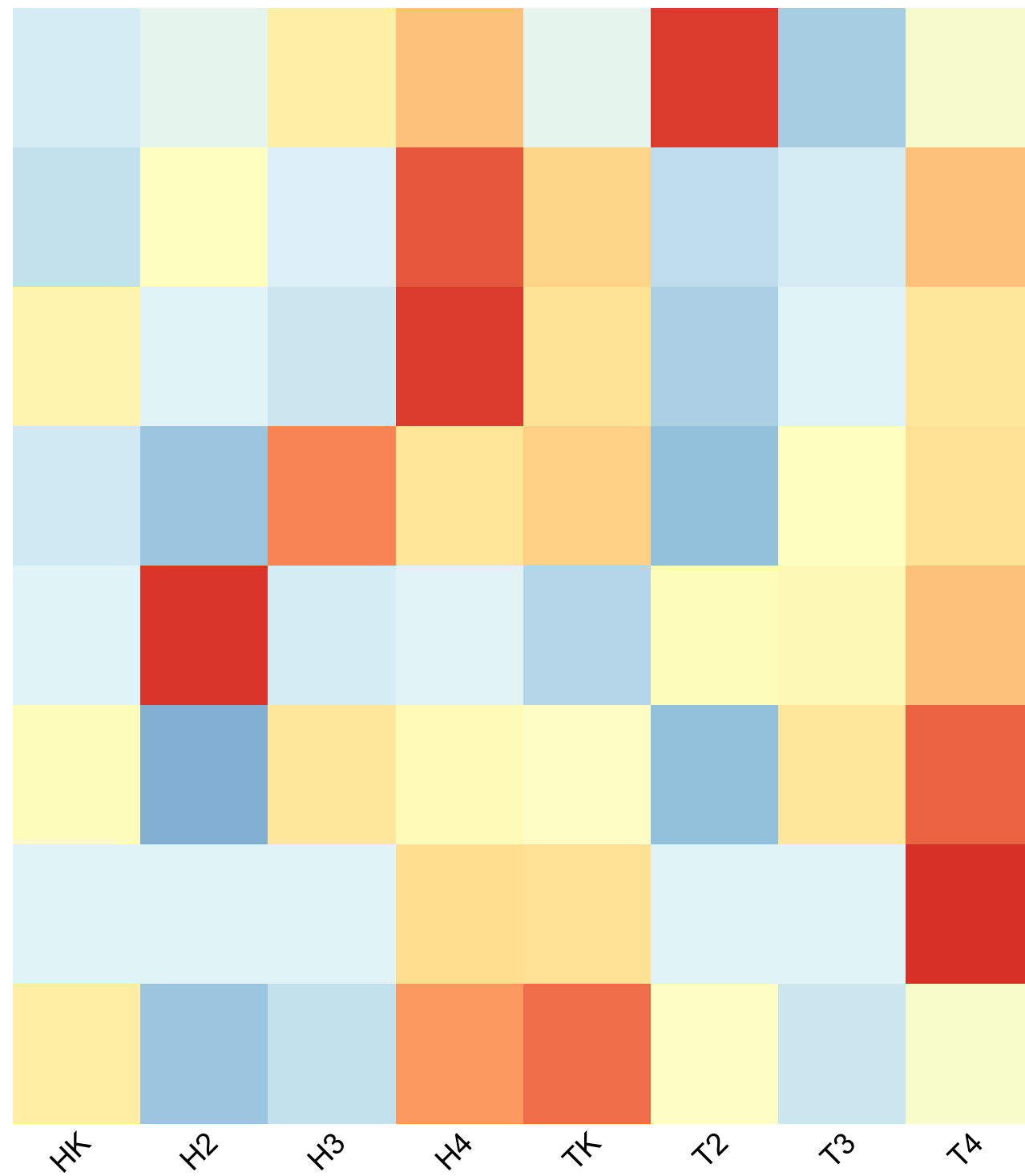

qPCR

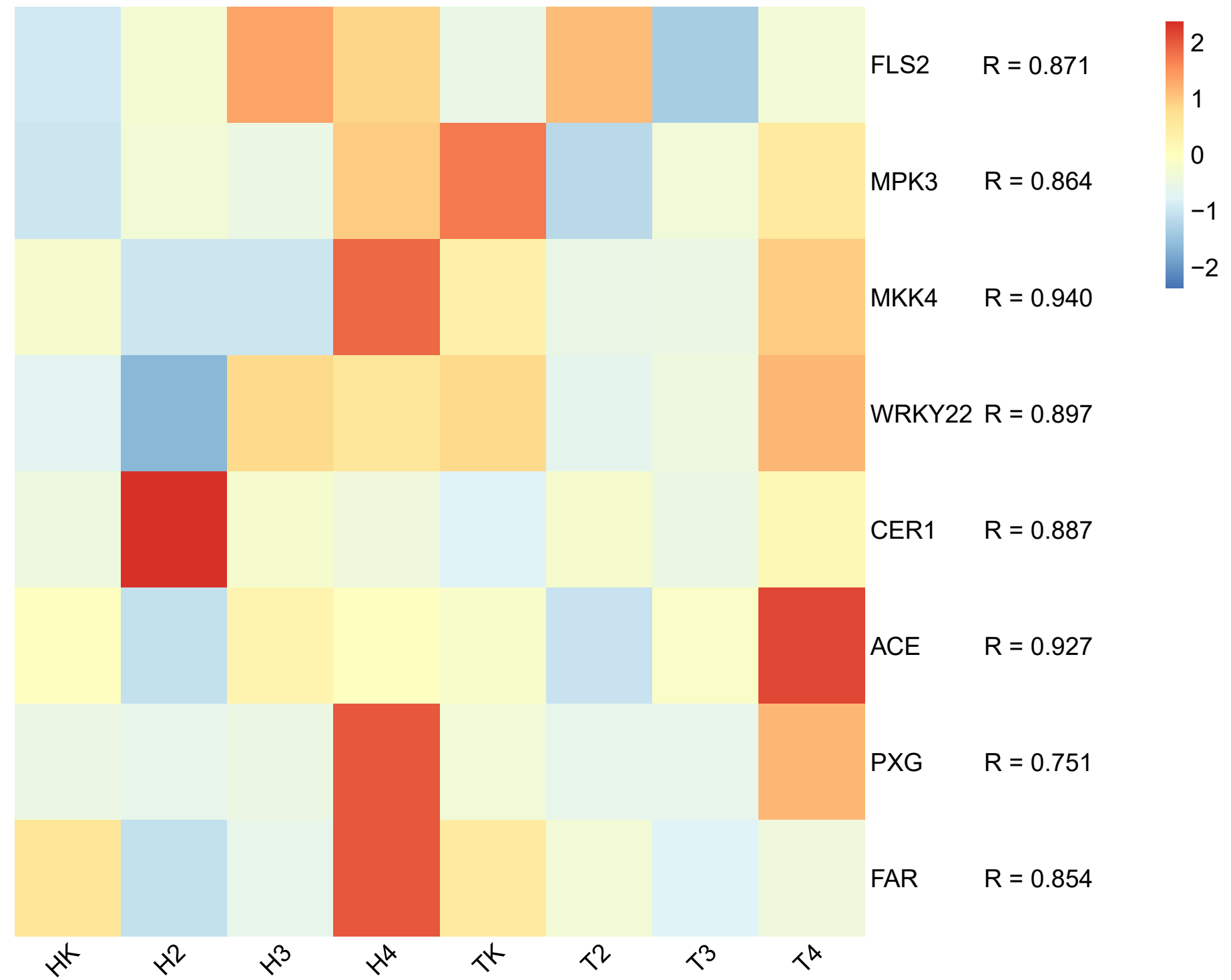

Supplement: Supplementary file 2 [file Image_2.pdf]
